# Supplementary material for: Brief empathy interventions online can decrease but not increase empathic tendencies
Source: Commun Psychol. 2025 Nov 14;3:157. doi: 10.1038/s44271-025-00364-w (PMC12618501; doi:10.1038/s44271-025-00364-w)
Supplement: Supplementary file 2 — Supplementary_Material_Brief empathy interventions online can decrease but not increase empathic tendencies [file 44271_2025_364_MOESM2_ESM.pdf]

Supplementary Material: Brief empathy interventions online can decrease but not increase empathic tendencies

Alexander Tagesson*[1 2], Annika Wallin[1], Philip Pärnamets[3]

1 Department of Philosophy, Division of Cognitive Science, Lund University, Lund, Sweden. **2** Agenda 2030 Graduate School, Lund University, Lund, Sweden. **3** Department of Clinical Neuroscience, Emotion Lab, Karolinska Institute, Stockholm, Sweden.

* alexander.tagesson@lucs.lu.se

Content:

1. Table SR1 – table showing all interventions; Table SR1.1 – table showing donation items; Table SR1.2 - table showing manipulation checks.
2. Study 1 – tables showing: participants' intervention ratings; model output & contrasts for all outcome measures; covariate analysis for donation & ymca
3. Study 2 – tables showing: participants' intervention ratings; model output & contrasts for all outcome measures; covariate analysis for donation & ymca
4. Study 3 – tables showing: participants' intervention ratings; model output & contrasts for all outcome measures; covariate analysis for donation & iac
5. Study 4 – tables showing: participants' intervention ratings; model output & contrasts for all outcome measures; covariate analysis for donation & iac
6. Study 5 - model output & contrasts for all outcome measures; covariate analysis for IRI subscales

Table SR1. Table showing all interventions and control used in this study.

Interventions	
Unlimited (study 1-4)	**Definition**: Empathy is defined as the ability to understand and share the feelings and thoughts of others. For example, empathizing with someone in distress involves understanding the situation from their perspective and feeling their negative emotions. Recent studies have found that *empathy is an unlimited resource*, so people *can feel* it toward a large number of people. Imagine that you are about to meet people in distress. Toward how many of them could you feel empathy? Please answer how many people you can empathize with: **From 0** – can't feel empathy toward anyone; **to 300** – can feel empathy toward three hundred people.
Limited (study 1-4)	**Definition**: Empathy is defined as the ability to understand and share the feelings and thoughts of others. For example, empathizing with someone in distress involves understanding the situation from their perspective and feeling their negative emotions. Recent studies have found that *empathy is a limited resource*, so people *cannot feel* it toward a large number of people. Imagine that you are about to meet people in distress. Toward how many of them could you feel empathy? Please answer how many people you can empathize with: **From 0** – can't feel empathy toward anyone; **to 3** – can feel empathy toward three people.
Malleable (study 1-3)	**Definition**: Empathy is defined as the ability to understand and share the feelings and thoughts of others. For example, empathizing with someone in distress involves understanding the situation from their perspective and feeling their negative emotions. Recent studies have shown that *we can regulate our empathy* and that it is not a rigid trait. That is, we can become more empathic if we try to empathize with people. Some of these studies also showed that when people learned that we can regulate our empathy, they put more effort into becoming more empathic. Imagine that you find yourself in a social situation where you would want to be empathic. How much would you be able to increase your empathy in that situation? Please answer: **From 0** – I would not be able to increase my empathy at all; **to 100** – I would be able to increase my empathy a lot.
Normative (study 1-3)	**Definition**: Empathy is defined as the ability to understand and share the feelings and thoughts of others. For example, empathizing with someone in distress involves understanding the situation from their perspective and feeling their negative emotions. Empathy is highly valued in most communities. Several studies demonstrate that people strongly value empathy and expect others in their community to be empathic. *Empathic people are also well-liked by their peers* because they better understand those around them. As people learn that their community values empathy, they often put more effort into relating to and understanding others. Imagine that you find

	yourself in a social situation where you would want to be empathic. How much would you be able to increase your empathy in that situation? Please answer: **From 0** – I would not be able to increase my empathy at all; **to 100** – I would be able to increase my empathy a lot.
Control (study 1-5)	Financial investments can be risky. On average, *people lose money when making stock investments*. A small percentage of stock investors make the largest gains, the rest often lose money on their investments. Some people can be so unfortunate that they lose much of their savings and sometimes find themselves in problematic financial situations. How risky do you think stock investments are? Please answer: **From 0** – Not risky at all; **to 100** – Very risky.
Normative (study 4)	**Definition:** Empathy is defined as the ability to understand and share the feelings and thoughts of others. For example, empathizing with someone in distress involves understanding the situation from their perspective and feeling their negative emotions. Empathy is highly valued in our community. Being empathic helps you understand other people. Several studies demonstrate that people strongly value empathy and expect both themselves and others in their community to be empathic. Imagine that you find yourself in a social situation where it is expected to be empathic in your community. How much would you be able to increase your empathy in that situation? Please answer: From 0 – I would not be able to increase my empathy at all; to 100 – I would be able to increase my empathy a lot.
Combo (study 4)	**Definition**: Empathy is defined as the ability to understand and share the feelings and thoughts of others. For example, empathizing with someone in distress involves understanding the situation from their perspective and feeling their negative emotions. Recent studies have shown that *we can regulate our empathy* and that it is not a rigid trait. That is, we can become more empathic if we try to empathize with people. Other studies demonstrate that people strongly value empathy, expect others in their community to be empathic and that *empathic people are well-liked by their peers*. Imagine that you find yourself in a social situation where you would want to be empathic. How much would you be able to increase your empathy in that situation? Please answer: **From 0** – I would not be able to increase my empathy at all; **to 100** – I would be able to increase my empathy a lot.
Unlimited (Study 5)	**Definition:** Empathy is defined as the ability to understand and share the feelings and thoughts of others. For example, empathizing with someone in distress involves understanding the situation from his/her perspective and feeling his/her negative emotions.<br>Apparently the idiom *"everyone has a place in my heart"* is scientifically correct: Studies have found that empathy is an unlimited resource so it *can* be felt toward a large number of people.
Limited (Study 5)	**Definition:** Empathy is defined as the ability to understand and share the feelings and thoughts of others. For example, empathizing with someone in distress involves understanding the situation from his/her perspective and feeling his/her negative emotions.<br>Apparently the idiom *"everyone has a place in my heart"* is scientifically incorrect: Studies have found that empathy is a limited resource so it *cannot* be felt toward a large number of people.
Hasson Control (Study 5)	**Definition:** Empathy is defined as the ability to understand and share the feelings and thoughts of others. For example, empathizing with someone in distress involves understanding the situation from his/her perspective and feeling his/her negative emotions.

Table SR1.1 Table showing full description of donation items used in all studies.

Donation item	

**YMCA (study 1-2)**	**Opportunity to donate to the YMCA**<br><br>You, and everyone else who participates in this experiment, will receive a bonus payment of 1 pound (100 pence). This bonus payment is an additional payment to the basic payment that you receive for participating in this experiment. Whatever you choose to do with your bonus payment, you will receive your basic payment for participating in this experiment.<br><br>As you know, Tyrone and people like him, receive help from the YMCA. You can choose to donate some, or all, of your bonus payment to the YMCA and thereby help people like Tyrone. **Note**, that whatever amount you choose to donate will be a real donation to the YMCA that the researchers conducting this study will make once it is completed.<br><br>Make your choice by using the slider below. Maximum amount that can be donated is 100 Pence. You can also choose to not donate any money to the YMCA by moving the cursor to 0. The amount you choose to donate will be deducted from your bonus payment.
**IAC (study 3-4)**	**Opportunity to donate to the Immigrants Assistance Center (IAC)**<br><br>You, and everyone else who participates in this experiment, will receive a bonus payment of 1 pound (100 pence). This bonus payment is an additional payment to the basic payment that you receive for participating in this experiment. Whatever you choose to do with your bonus payment, you will receive your basic payment for participating in this experiment.<br><br>People that migrate to the US are sometimes in need of help to cover basic needs, such as obtaining proper clothes, food and hygiene products. You can choose to donate some, or all, of your bonus payment to the Immigrants Assistance Center (IAC), which help people that migrated to the US with basic needs. **Note**, that whatever amount you choose to donate will be a real donation to the IAC that the researchers conducting this study will make once it is completed.<br><br>Make your choice by using the slider below. Maximum amount that can be donated is 100 pence. You can also choose to not donate any money to the IAC by moving the cursor to 0. The amount you choose to donate will be deducted from your bonus payment.

Table SR1.3 Full description of the manipulation checks used in studies 1-5.

	Studies				
Manipulation checks	1	2	3	4	5
To what extent do you think that empathy is a limited resource, for example if you feel a lot of empathy with one person, you will not be able to feel as much empathy with another person?) - From 1 – I think empathy is a very limited resource; to 7 – I do not think empathy is a limited	X	X	X	Na	Na

resource at all (manipulation check for Unlimited and Limited).					
To what extent do you think we can change our ability to be empathic? - From 1 – I do not think that we can change our ability to be empathic at all; to 7 – I think we can change our ability to be empathic a lot (manipulation check for Malleable in studies 1-2 and for Combo in study 4).	X	X	Na	X	Na
After participating in this experiment and learning more about empathy, how motivated do you feel to try to increase the empathy you feel in your everyday life? - From 1 – I do not feel motivated at all; to 7 – I feel very motivated (manipulation check for Normative).	X	X	Na	Na	Na
People have an infinite amount of empathy at their disposal - From 1 – strongly disagree; to – strongly agree (manipulation check for Unlimited and Limited).	Na	Na	Na	X	X
There is no limit to the extent of empathy a person can feel - From 1 – strongly disagree; to – strongly agree (manipulation check for Unlimited and Limited).	Na	Na	Na	X	X
There is a limit to how much empathy we can feel towards others - From 1 – strongly disagree; to – strongly agree (manipulation check for Unlimited and Limited, reverse scored when analysed).	Na	Na	Na	X	X
One cannot be empathetic with everyone - From 1 – strongly disagree; to – strongly agree (manipulation check for Unlimited and Limited, reverse scored when analysed).	Na	Na	Na	X	X
Do you agree or disagree with the view that people should strive to be empathic?<br>From 1 – Strongly disagree; to 7 – Strongly agree (manipulation check for Normative in study 4)	Na	Na	Na	X	Na

Study 1

Table SR2.1: Participants' Intervention ratings in Study 1

Condition	*Mean (SD)*
Normative	73.19 (22.11)
Unlimited	31.47 (88.06)
Limited	2.41 (0.77)
Malleable	67.23 (24.08)
Control	70.86 (18.95)

Manipulation checks

Unlimited belief

Table SR2.2. Model output from multiple regression model for beliefs that empathy is an un/limited resource

condition	emmean	lower.HPD	upper.HPD
Control	0.025	-0.126	0.178
Limited	-0.218	-0.376	-0.059
Malleable	-0.037	-0.204	0.126
Normative	-0.020	-0.182	0.139
Unlimited	0.257	0.096	0.420

Table SR2.3. Contrasts between model coefficients from multiple regression models for beliefs that empathy is an un/limited resource

Hypothesis	Estimate	Est.Error	CI.Lower	CI.Upper	Evid.Ratio	Post.Prob
Limited - Control	-0.243	0.111	-0.463	-0.025	1.147	0.534
Unlimited - Control	0.232	0.114	0.010	0.454	1.512	0.602
Malleable - Control	-0.063	0.114	-0.289	0.160	10.396	0.912
Normative - Control	-0.046	0.112	-0.265	0.176	11.217	0.918
Unlimited - Limited	0.475	0.115	0.249	0.703	0.004	0.004
Malleable - Limited	0.180	0.116	-0.050	0.403	3.673	0.786
Normative - Limited	0.197	0.114	-0.025	0.422	3.048	0.753
Unlimited - Malleable	0.295	0.118	0.064	0.528	0.478	0.323
Normative - Malleable	0.018	0.117	-0.212	0.249	11.570	0.920
Unlimited - Normative	0.277	0.115	0.054	0.505	0.631	0.387

Malleability belief

Table SR2.4 Model output from multiple regression model for beliefs that empathy is malleable

condition	emmean	lower.HPD	upper.HPD
Control	-0.069	-0.223	0.086

Limited	-0.031	-0.197	0.128
Malleable	0.105	-0.053	0.279
Normative	0.027	-0.137	0.184
Unlimited	-0.018	-0.181	0.138

Table SR2.5 Contrasts between model coefficients from multiple regression models for beliefs that empathy is malleable

Hypothesis	Estimate	Est.Error	CI.Lower	CI.Upper	Evid.Ratio	Post.Prob
Limited - Control	0.037	0.113	-0.185	0.260	11.94	0.923
Unlimited - Control	0.050	0.113	-0.172	0.272	11.18	0.918
Malleable - Control	0.173	0.116	-0.052	0.401	4.19	0.807
Normative - Control	0.094	0.113	-0.126	0.316	8.87	0.899
Unlimited - Limited	0.014	0.117	-0.215	0.242	11.68	0.921
Malleable - Limited	0.137	0.118	-0.098	0.369	6.12	0.860
Normative - Limited	0.058	0.116	-0.171	0.284	10.73	0.915
Unlimited - Malleable	-0.123	0.118	-0.355	0.108	6.98	0.875
Normative - Malleable	-0.079	0.117	-0.310	0.150	9.71	0.907
Unlimited - Normative	-0.044	0.116	-0.272	0.185	11.60	0.921

Normative belief

Table SR2.6 Model output from multiple regression model for beliefs that empathy is normative

condition	emmean	lower.HPD	upper.HPD
Control	-0.107	-0.257	0.050
Limited	-0.042	-0.201	0.121
Malleable	0.021	-0.144	0.192
Normative	0.057	-0.102	0.213
Unlimited	0.091	-0.079	0.251

Table SR2.7. Contrasts between model coefficients from multiple regression models for beliefs that empathy is normative

Hypothesis	Estimate	Est.Error	CI.Lower	CI.Upper	Evid.Ratio	Post.Prob
Limited - Control	0.065	0.114	-0.156	0.290	10.76	0.915
Unlimited - Control	0.197	0.115	-0.031	0.423	2.79	0.736
Malleable - Control	0.129	0.116	-0.098	0.357	6.96	0.874
Normative - Control	0.163	0.113	-0.059	0.386	4.54	0.819
Unlimited - Limited	0.133	0.117	-0.095	0.361	6.38	0.865
Malleable - Limited	0.064	0.118	-0.169	0.295	10.72	0.915
Normative - Limited	0.099	0.116	-0.131	0.326	8.47	0.894
Unlimited - Malleable	0.069	0.119	-0.165	0.301	9.98	0.909

Normative - Malleable	0.035	0.118	-0.198	0.267	11.69	0.921
Unlimited - Normative	0.034	0.118	-0.196	0.264	11.33	0.919

Empathy

Table SR2.8 Model output from multiple regression model for single-item empathy

condition	emmean	lower.HPD	upper.HPD
Control	0.089	-0.066	0.244
Limited	-0.196	-0.356	-0.035
Malleable	-0.017	-0.186	0.150
Normative	0.052	-0.109	0.211
Unlimited	0.066	-0.098	0.224

Table SR2.9. Contrasts between model coefficients from multiple regression models for single-item empathy

Hypothesis	Estimate	Est.Error	CI.Lower	CI.Upper	Evid.Ratio	Post.Prob
Limited - Control	-0.285	0.114	-0.506	-0.061	0.567	0.362
Unlimited - Control	-0.023	0.114	-0.246	0.202	12.711	0.927
Malleable - Control	-0.106	0.117	-0.336	0.124	8.020	0.889
Normative - Control	-0.037	0.113	-0.255	0.183	11.977	0.923
Unlimited - Limited	0.261	0.116	0.036	0.487	0.992	0.498
Malleable - Limited	0.178	0.118	-0.051	0.408	3.977	0.799
Normative - Limited	0.248	0.116	0.020	0.475	1.272	0.560
Unlimited - Malleable	0.083	0.118	-0.148	0.316	9.554	0.905
Normative - Malleable	0.069	0.119	-0.166	0.301	10.404	0.912

Unlimited - Normative	0.014	0.117	-0.215	0.245	11.979	0.923

Empathic reactions

Table SR2.10 Model output from multiple regression model for empathic reactions

condition	emmean	lower.HPD	upper.HPD
Control	0.100	-0.051	0.255
Limited	-0.153	-0.307	0.009
Malleable	-0.044	-0.209	0.118
Normative	0.028	-0.127	0.191
Unlimited	0.065	-0.099	0.229

Table SR2.11. Contrasts between model coefficients from multiple regression models for empathic reactions

Hypothesis	Estimate	Est.Error	CI.Lower	CI.Upper	Evid.Ratio	Post.Prob
Limited - Control	-0.253	0.113	-0.474	-0.033	0.995	0.499
Unlimited - Control	-0.035	0.114	-0.262	0.189	12.056	0.923
Malleable - Control	-0.144	0.115	-0.369	0.082	5.736	0.852
Normative - Control	-0.071	0.113	-0.293	0.152	10.151	0.910
Unlimited - Limited	0.218	0.117	-0.013	0.447	2.229	0.690
Malleable - Limited	0.109	0.116	-0.118	0.335	7.922	0.888
Normative - Limited	0.181	0.115	-0.045	0.405	3.669	0.786
Unlimited - Malleable	0.109	0.119	-0.123	0.340	7.922	0.888
Normative - Malleable	0.073	0.118	-0.157	0.302	10.032	0.909
Unlimited - Normative	0.036	0.118	-0.194	0.263	11.588	0.921

Empathic drivers

Table SR2.12 Model output from multiple regression model for empathic drivers

condition	emmean	lower.HPD	upper.HPD
Control	-0.034	-0.191	0.115
Limited	-0.109	-0.275	0.048
Malleable	-0.003	-0.171	0.158
Normative	0.121	-0.038	0.278
Unlimited	0.033	-0.130	0.196

Table SR2.13. Contrasts between model coefficients from multiple regression models for empathic drivers

Hypothesis	Estimate	Est.Error	CI.Lower	CI.Upper	Evid.Ratio	Post.Prob
Limited - Control	-0.075	0.113	-0.298	0.147	10.00	0.909
Unlimited - Control	0.067	0.115	-0.157	0.290	10.38	0.912
Malleable - Control	0.031	0.115	-0.197	0.259	12.23	0.924
Normative - Control	0.155	0.114	-0.067	0.377	5.05	0.835
Unlimited - Limited	0.142	0.117	-0.086	0.371	5.85	0.854
Malleable - Limited	0.105	0.117	-0.124	0.336	8.36	0.893
Normative - Limited	0.230	0.115	0.006	0.459	1.63	0.620
Unlimited - Malleable	0.037	0.119	-0.197	0.268	11.44	0.920
Normative - Malleable	0.125	0.116	-0.105	0.350	7.03	0.875
Unlimited - Normative	-0.088	0.117	-0.317	0.142	9.39	0.904

Donation

Table SR2.14 Model output from multiple regression model for donations

condition	emmean	lower.HPD	upper.HPD
Control	-0.021	-0.177	0.134
Limited	-0.036	-0.196	0.122
Malleable	-0.025	-0.189	0.144
Normative	0.127	-0.034	0.284
Unlimited	-0.046	-0.205	0.122

Table SR2.15. Contrasts between model coefficients from multiple regression models for donations

Hypothesis	Estimate	Est.Error	CI.Lower	CI.Upper	Evid.Ratio	Post.Prob
Limited - Control	-0.014	0.113	-0.238	0.209	12.28	0.925
Unlimited - Control	-0.025	0.115	-0.250	0.202	12.25	0.925
Malleable - Control	-0.004	0.116	-0.234	0.226	12.33	0.925
Normative - Control	0.148	0.114	-0.075	0.369	5.38	0.843
Unlimited - Limited	-0.011	0.117	-0.242	0.219	12.35	0.925
Malleable - Limited	0.010	0.117	-0.220	0.240	12.17	0.924
Normative - Limited	0.162	0.116	-0.066	0.389	4.51	0.819
Unlimited - Malleable	-0.021	0.119	-0.256	0.215	11.45	0.920

Normative - Malleable	0.152	0.119	-0.083	0.385	5.26	0.840
Unlimited - Normative	-0.172	0.116	-0.399	0.056	4.09	0.804

Covariate YMCA

Table SR2.16. Summarized model output for multiple regression covariate model with an interaction between condition and ymca

	Estimate	Est.Error	l-95% CI	u-95% CI	Rhat	Bulk_ESS
Control	0.01	0.07	-0.13	0.16	1.00	35824
Limited	-0.03	0.08	-0.18	0.12	1.00	33258
Malleable	-0.06	0.08	-0.21	0.10	1.00	32368
Normative	0.11	0.08	-0.04	0.26	1.00	32564
Unlimited	-0.04	0.08	-0.19	0.11	1.00	35223
YMCA	0.38	0.07	0.24	0.52	1.00	11059
Limited:YMCA	-0.11	0.11	-0.33	0.10	1.00	15430
Malleable:YMCA	0.05	0.11	-0.16	0.26	1.00	15698
Normative:YMCA	-0.06	0.11	-0.27	0.14	1.00	15237
Unlimited:YMCA	-0.05	0.10	-0.24	0.15	1.00	14832

Study 2

Table SR3.1. Participants' intervention ratings in Study 2

Condition	*Mean (SD)*
Normative	71.36 (21.54)

Unlimited	222.39 (90.84)
Limited	2.47 (0.74)
Malleable	71.36 (22.26)
Control	69.16 (18.31)

Manipulation checks

Unlimited belief

Table SR3.2 Model output from multiple regression model for the belief that empathy is an un/limited resource

condition	emmean	lower.HPD	upper.HPD
Control	0.051	-0.112	0.210
Limited	-0.220	-0.384	-0.054
Malleable	0.082	-0.077	0.245
Normative	-0.072	-0.231	0.081
Unlimited	0.153	0.000	0.312

Table SR3.3. Contrasts between model coefficients from multiple regression models for beliefs about empathy being an un/limited resource

Hypothesis	Estimate	Est.Error	CI.Lower	CI.Upper	Evid.Ratio	Post.Prob
Limited - Control	-0.271	0.117	-0.501	-0.042	0.873	0.466
Unlimited - Control	0.102	0.115	-0.125	0.327	8.278	0.892
Malleable - Control	0.031	0.116	-0.198	0.257	11.885	0.922
Normative - Control	-0.123	0.115	-0.350	0.106	7.094	0.876
Unlimited - Limited	0.372	0.115	0.147	0.596	0.089	0.082
Malleable - Limited	0.301	0.118	0.072	0.531	0.457	0.314
Normative - Limited	0.147	0.116	-0.078	0.373	5.870	0.854

Unlimited - Malleable	0.071	0.114	-0.152	0.294	10.293	0.911
Normative - Malleable	-0.154	0.115	-0.378	0.071	5.222	0.839
Unlimited - Normative	0.225	0.113	0.002	0.446	1.730	0.634

Malleability belief

Table SR3.4 Model output from multiple regression model for the belief that empathy is malleable

**condition**	**emmean**	**lower.HPD**	**upper.HPD**
Control	0.019	-0.146	0.176
Limited	-0.106	-0.271	0.054
Malleable	0.175	0.015	0.334
Normative	0.081	-0.076	0.233
Unlimited	-0.153	-0.308	0.004

Table SR3.5. Contrasts between model coefficients from multiple regression models for beliefs about empathy being malleable

**Hypothesis**	**Estimate**	**Est.Error**	**CI.Lower**	**CI.Upper**	**Evid.Ratio**	**Post.Prob**
Limited - Control	-0.125	0.116	-0.355	0.103	6.986	0.875
Unlimited - Control	-0.172	0.114	-0.396	0.049	3.951	0.798
Malleable - Control	0.155	0.115	-0.069	0.379	5.164	0.838
Normative - Control	0.061	0.113	-0.159	0.286	11.021	0.917
Unlimited - Limited	-0.047	0.115	-0.273	0.183	11.380	0.919

Malleable - Limited	0.280	0.116	0.053	0.506	0.671	0.401
Normative - Limited	0.186	0.115	-0.039	0.409	3.216	0.763
Unlimited - Malleable	-0.328	0.113	-0.547	-0.108	0.185	0.156
Normative - Malleable	-0.094	0.114	-0.316	0.130	8.806	0.898
Unlimited - Normative	-0.234	0.112	-0.454	-0.016	1.357	0.576

Normative belief

Table SR3.6 Model output from multiple regression model for the belief that empathy is normative

**condition**	**emmean**	**lower.HPD**	**upper.HPD**
Control	0.041	-0.114	0.211
Limited	-0.034	-0.195	0.130
Malleable	0.008	-0.150	0.170
Normative	0.099	-0.058	0.256
Unlimited	-0.106	-0.263	0.053

Table SR3.7. Contrasts between model coefficients from multiple regression models for beliefs about empathy being normative

**Hypothesis**	**Estimate**	**Est.Error**	**CI.Lower**	**CI.Upper**	**Evid.Ratio**	**Post.Prob**
Limited - Control	-0.075	0.117	-0.307	0.156	10.20	0.911
Unlimited - Control	-0.147	0.116	-0.375	0.080	5.61	0.849
Malleable - Control	-0.032	0.116	-0.264	0.194	12.08	0.924
Normative - Control	0.058	0.115	-0.168	0.283	11.07	0.917

Unlimited - Limited	-0.072	0.116	-0.298	0.157	10.18	0.911
Malleable - Limited	0.043	0.117	-0.189	0.271	11.44	0.920
Normative - Limited	0.133	0.116	-0.097	0.357	6.72	0.870
Unlimited - Malleable	-0.114	0.116	-0.342	0.115	7.78	0.886
Normative - Malleable	0.090	0.116	-0.136	0.317	9.26	0.903
Unlimited - Normative	-0.204	0.114	-0.427	0.021	2.54	0.718

Empathy

Table SR3.8 Model output from multiple regression model for single-item empathy

**condition**	**emmean**	**lower.HPD**	**upper.HPD**
Control	0.109	-0.052	0.269
Limited	-0.124	-0.285	0.041
Malleable	-0.022	-0.187	0.137
Normative	0.058	-0.097	0.214
Unlimited	-0.025	-0.182	0.129

Table SR3.9. Contrasts between model coefficients from multiple regression models for single-item empathy

**Hypothesis**	**Estimate**	**Est.Error**	**CI.Lower**	**CI.Upper**	**Evid.Ratio**	**Post.Prob**
Limited - Control	-0.233	0.116	-0.461	-0.003	1.70	0.629
Unlimited - Control	-0.134	0.115	-0.361	0.089	6.54	0.867

Malleable - Control	-0.132	0.116	-0.360	0.095	6.54	0.867
Normative - Control	-0.050	0.115	-0.275	0.174	11.02	0.917
Unlimited - Limited	0.099	0.116	-0.130	0.326	8.80	0.898
Malleable - Limited	0.101	0.118	-0.131	0.330	8.41	0.894
Normative - Limited	0.182	0.115	-0.041	0.409	3.49	0.777
Unlimited - Malleable	-0.002	0.115	-0.229	0.222	12.61	0.927
Normative - Malleable	0.081	0.115	-0.142	0.306	9.49	0.905
Unlimited - Normative	-0.084	0.113	-0.306	0.138	10.01	0.909

Empathic reactions

Table SR3.10 Model output from multiple regression model for empathic reactions

condition	emmean	lower.HPD	upper.HPD
Control	0.129	-0.037	0.287
Limited	-0.091	-0.253	0.070
Malleable	-0.042	-0.199	0.125
Normative	0.066	-0.092	0.219
Unlimited	-0.049	-0.210	0.104

Table SR3.11. Contrasts between model coefficients from multiple regression models for empathic reactions

Hypothesis	Estimate	Est.Error	CI.Lower	CI.Upper	Evid.Ratio	Post.Prob	Star
Limited - Control	-0.220	0.116	-0.447	0.008	1.96	0.662	
Unlimited - Control	-0.179	0.116	-0.406	0.049	3.82	0.793	

Malleable - Control	-0.171	0.117	-0.400	0.060	4.14	0.806	
Normative - Control	-0.063	0.115	-0.286	0.163	10.65	0.914	
Unlimited - Limited	0.041	0.116	-0.188	0.270	11.43	0.920	
Malleable - Limited	0.049	0.118	-0.181	0.278	11.10	0.917	
Normative - Limited	0.157	0.115	-0.068	0.383	5.14	0.837	
Unlimited - Malleable	-0.008	0.116	-0.234	0.219	11.86	0.922	
Normative - Malleable	0.108	0.115	-0.116	0.330	7.90	0.888	
Unlimited - Normative	-0.116	0.113	-0.337	0.105	7.42	0.881	

Empathic drivers

Table SR3.12 Model output from multiple regression model for empathic drivers

**condition**	**emmean**	**lower.HPD**	**upper.HPD**
Control	0.033	-0.130	0.194
Limited	-0.004	-0.169	0.165
Malleable	-0.034	-0.199	0.124
Normative	0.055	-0.098	0.213
Unlimited	-0.044	-0.207	0.113

Table SR3.13. Contrasts between model coefficients from multiple regression models for empathic drivers

Hypothesis	Estimate	Est.Error	CI.Lower	CI.Upper	Evid.Ratio	Post.Prob
Limited - Control	-0.037	0.119	-0.271	0.192	11.71	0.921
Unlimited - Control	-0.078	0.116	-0.309	0.151	9.82	0.908
Malleable - Control	-0.068	0.117	-0.298	0.160	10.67	0.914
Normative - Control	0.022	0.116	-0.204	0.248	11.84	0.922
Unlimited - Limited	-0.041	0.118	-0.272	0.192	11.24	0.918
Malleable - Limited	-0.031	0.118	-0.264	0.200	11.67	0.921
Normative - Limited	0.059	0.117	-0.171	0.289	10.60	0.914
Unlimited - Malleable	-0.011	0.116	-0.239	0.214	12.22	0.924
Normative - Malleable	0.090	0.115	-0.136	0.316	9.27	0.903
Unlimited - Normative	-0.101	0.114	-0.324	0.121	8.73	0.897

Donation

Table SR3.14 Model output from multiple regression model for donations

condition	emmean	lower.HPD	upper.HPD
Control	0.090	-0.074	0.249
Limited	-0.172	-0.339	-0.009

Malleable	-0.045	-0.206	0.114
Normative	0.056	-0.101	0.211
Unlimited	0.058	-0.106	0.215

Table SR3.15. Contrasts between model coefficients from multiple regression models for donations

Hypothesis	Estimate	Est.Error	CI.Lower	CI.Upper	Evid.Ratio	Post.Prob
Limited - Control	-0.262	0.118	-0.492	-0.031	1.05	0.511
Unlimited - Control	-0.032	0.117	-0.262	0.195	11.74	0.921
Malleable - Control	-0.136	0.116	-0.365	0.092	6.32	0.863
Normative - Control	-0.035	0.115	-0.264	0.190	11.79	0.922
Unlimited - Limited	0.230	0.117	0.000	0.460	1.86	0.651
Malleable - Limited	0.127	0.118	-0.105	0.355	6.88	0.873
Normative - Limited	0.228	0.116	0.000	0.453	1.83	0.647
Unlimited - Malleable	0.103	0.115	-0.123	0.328	8.47	0.894
Normative - Malleable	0.101	0.114	-0.125	0.326	8.30	0.892
Unlimited - Normative	0.002	0.114	-0.220	0.226	12.77	0.927

covariate YMCA

Table SR3.16. Summarizing model output for multiple regression model with interaction between condition and covariate ymca

	Estimate	Est.Error	l-95% CI	u-95% CI	Rhat	Bulk_ESS
Control	0.09	0.08	-0.06	0.25	1.00	29987
Limited	-0.17	0.08	-0.32	-0.01	1.00	30264

Malleable	-0.01	0.08	-0.17	0.14	1.00	28124
Normative	0.04	0.08	-0.10	0.19	1.00	29722
Unlimited	0.03	0.08	-0.12	0.18	1.00	31086
YMCA	0.37	0.08	0.22	0.53	1.00	13522
Limited:YMCA	-0.14	0.11	-0.35	0.07	1.00	16818
Malleable:YMCA	-0.06	0.11	-0.27	0.15	1.00	16846
Normative:YMCA	0.04	0.11	-0.18	0.25	1.00	17276
Unlimited:YMCA	-0.09	0.12	-0.32	0.14	1.00	18320

Study 3

Table SR4.1. Participants' intervention ratings in Study 3

Condition	*Mean (SD)*
Normative	75.14 (21.54)
Unlimited	240.43 (90.84)
Limited	2.54 (0.74)
Malleable	70.72 (22.26)
Control	69.76 (18.31)

Manipulation check (Unlimited belief)

Table SR4.2 Model output from multiple regression model for belief that empathy is an un/limited resource

condition	emmean	lower.HPD	upper.HPD
Control	-0.042	-0.108	0.025
Limited	-0.038	-0.106	0.029
Malleable	-0.130	-0.198	-0.064
Normative	0.027	-0.037	0.094

Unlimited	0.184	0.117	0.252

Table SR4.3 Contrasts between model coefficients from multiple regression models for beliefs about empathy being an un/limited resource

Hypothesis	Estimate	Est.Error	CI.Lower	CI.Upper	Evid.Ratio	Post.Prob
Limited - Control	0.004	0.048	-0.091	0.099	29.076	0.967
Unlimited - Control	0.226	0.049	0.130	0.321	0.000	0.000
Malleable - Control	-0.088	0.049	-0.183	0.008	5.898	0.855
Normative - Control	0.069	0.048	-0.024	0.163	10.081	0.910
Unlimited - Limited	0.222	0.049	0.127	0.318	0.000	0.000
Malleable - Limited	-0.092	0.048	-0.185	0.004	5.114	0.836
Normative - Limited	0.066	0.048	-0.028	0.161	11.469	0.920
Unlimited - Malleable	0.314	0.049	0.218	0.410	0.000	0.000
Normative - Malleable	0.157	0.048	0.063	0.251	0.117	0.105
Unlimited - Normative	0.156	0.048	0.062	0.251	0.139	0.122

Empathy

Table SR4.4 Model output from multilevel regression model for single-item empathy

condition	emmean	lower.HPD	upper.HPD
Control	-0.010	-0.165	0.158
Limited	-0.067	-0.227	0.092
Malleable	-0.089	-0.247	0.067
Normative	0.086	-0.069	0.244
Unlimited	0.079	-0.075	0.241

Table SR4.5 Contrasts between model coefficients from multilevel regression models for empathy

Hypothesis	Estimate	Est.Error	CI.Lower	CI.Upper	Evid.Ratio	Post.Prob
Limited - Control	-0.057	0.092	-0.237	0.123	12.78	0.927
Unlimited - Control	0.090	0.093	-0.094	0.272	9.48	0.905
Malleable - Control	-0.080	0.092	-0.261	0.098	10.29	0.911
Normative - Control	0.096	0.090	-0.083	0.271	8.80	0.898
Unlimited - Limited	0.147	0.092	-0.030	0.326	4.50	0.818
Malleable - Limited	-0.023	0.092	-0.205	0.157	16.10	0.942
Normative - Limited	0.153	0.090	-0.025	0.330	3.59	0.782
Unlimited - Malleable	0.170	0.093	-0.011	0.355	3.00	0.750
Normative - Malleable	0.176	0.090	0.000	0.355	2.38	0.704
Unlimited - Normative	-0.007	0.090	-0.185	0.169	15.52	0.939

Empathic reactions

Table SR4.6 Model output from multilevel regression model for empathic reactions

condition	emmean	lower.HPD	upper.HPD
Control	-0.010	-0.165	0.158
Limited	-0.067	-0.227	0.092
Malleable	-0.089	-0.247	0.067

Normative	0.086	-0.069	0.244
Unlimited	0.079	-0.075	0.241

Table SR4.7 Contrasts between model coefficients from multilevel regression model for empathic reactions

Hypothesis	Estimate	Est.Error	CI.Lower	CI.Upper	Evid.Ratio	Post.Prob
Limited - Control	-0.057	0.092	-0.237	0.123	12.59	0.926
Unlimited - Control	0.090	0.093	-0.094	0.272	9.38	0.904
Malleable - Control	-0.080	0.092	-0.261	0.098	10.32	0.912
Normative - Control	0.096	0.090	-0.083	0.271	8.85	0.898
Unlimited - Limited	0.147	0.092	-0.030	0.326	4.40	0.815
Malleable - Limited	-0.023	0.092	-0.205	0.157	15.56	0.940
Normative - Limited	0.153	0.090	-0.025	0.330	3.55	0.780
Unlimited - Malleable	0.170	0.093	-0.011	0.355	3.04	0.752
Normative - Malleable	0.176	0.090	0.000	0.355	2.33	0.700
Unlimited - Normative	-0.007	0.090	-0.185	0.169	15.03	0.938

Empathic drivers

Table SR4.6 Model output from multilevel regression model for empathic reactions

condition	emmean	lower.HPD	upper.HPD
Control	-0.154	-0.223	-0.087

Limited	0.041	-0.028	0.108
Malleable	0.013	-0.055	0.078
Normative	0.113	0.047	0.178
Unlimited	-0.017	-0.084	0.053

Table SR4.7 Contrasts between model coefficients from multilevel regression model for empathic reactions

Hypothesis	Estimate	Est.Error	CI.Lower	CI.Upper	Evid.Ratio	Post.Prob
Limited - Control	0.195	0.049	0.098	0.290	0.017	0.016
Unlimited - Control	0.138	0.049	0.041	0.234	0.623	0.384
Malleable - Control	0.167	0.049	0.073	0.262	0.120	0.107
Normative - Control	0.267	0.048	0.173	0.362	0.000	0.000
Unlimited - Limited	-0.057	0.049	-0.153	0.040	14.233	0.934
Malleable - Limited	-0.028	0.049	-0.125	0.067	24.389	0.961
Normative - Limited	0.072	0.048	-0.023	0.167	9.065	0.901
Unlimited - Malleable	-0.029	0.049	-0.126	0.068	24.530	0.961
Normative - Malleable	0.100	0.048	0.005	0.194	3.560	0.781
Unlimited - Normative	-0.130	0.049	-0.225	-0.034	0.874	0.466

Donation

Table SR4.10 Model output from multiple regression model for donations

condition	emmean	lower.HPD	upper.HPD
Control	-0.057	-0.123	0.011
Limited	0.022	-0.044	0.090

Malleable	-0.006	-0.075	0.059
Normative	0.088	0.022	0.155
Unlimited	-0.052	-0.120	0.016

Table SR4.11 Contrasts between model coefficients from multiple regression models for donations

Hypothesis	Estimate	Est.Error	CI.Lower	CI.Upper	Evid.Ratio	Post.Prob
Limited - Control	0.079	0.049	-0.015	0.173	8.181	0.891
Unlimited - Control	0.004	0.049	-0.092	0.100	29.327	0.967
Malleable - Control	0.050	0.048	-0.045	0.146	16.711	0.944
Normative - Control	0.145	0.048	0.049	0.239	0.265	0.209
Unlimited - Limited	-0.074	0.049	-0.171	0.022	9.367	0.904
Malleable - Limited	-0.029	0.048	-0.124	0.066	23.782	0.960
Normative - Limited	0.066	0.048	-0.029	0.161	11.896	0.922
Unlimited - Malleable	-0.046	0.049	-0.141	0.049	19.490	0.951
Normative - Malleable	0.095	0.048	0.000	0.189	4.600	0.821
Unlimited - Normative	-0.140	0.049	-0.237	-0.046	0.468	0.319

Covariate IAC

Table SR4.12 Summarized model output for multiple regression model with an interaction between condition and IAC

	Estimate	Est.Error	l-95% CI	u-95% CI	Rhat	Bulk_ESS
Control	-0.03	0.03	-0.09	0.04	1.00	30409

Limited	0.01	0.03	-0.05	0.07	1.00	32211
Malleable	-0.02	0.03	-0.08	0.04	1.00	32440
Normative	0.06	0.03	0.00	0.12	1.00	32081
Unlimited	-0.03	0.03	-0.09	0.03	1.00	33129
IAC	0.45	0.03	0.39	0.51	1.00	12457
Limited:IAC	-0.16	0.04	-0.25	-0.07	1.00	15793
Malleable:IAC	-0.05	0.05	-0.14	0.04	1.00	16424
Normative:IAC	-0.02	0.05	-0.11	0.07	1.00	17014
Unlimited:IAC	-0.07	0.04	-0.16	0.01	1.00	15673

Study 4

Table SR5.1. Participants' intervention ratings in Study 4

Condition	***Mean (SD)***
Unlimited	228.4 (85.5)
Limited	2.5 (0.6)
Combo	73.8 (19.8)
Normative	75.0 (21.9)
Control	69.5 (20.5)

Manipulation checks

Unlimited belief

Table SR5.2 Model output from multiple regression model for belief that empathy is an un/limited resource

**condition**	**emmean**	**lower.HPD**	**upper.HPD**
Combo	-0.144	-0.261	-0.032
Control	-0.080	-0.191	0.041

Limited	-0.102	-0.217	0.014
Normative	-0.061	-0.179	0.054
Unlimited	0.392	0.271	0.504

Table SR5.3 Contrasts between model coefficients from multiple regression models for beliefs about empathy being an un/limited resource

Hypothesis	Estimate	Est.Error	CI.Lower	CI.Upper	Evid.Ratio	Post.Prob
Limited - Control	-0.022	0.084	-0.184	0.143	3.42	0.774
Unlimited - Control	0.473	0.084	0.309	0.639	0.00	0.000
Combo - Control	-0.065	0.084	-0.230	0.101	2.54	0.718
Normative - Control	0.020	0.086	-0.146	0.194	3.16	0.760
Unlimited - Limited	0.495	0.084	0.331	0.659	0.00	0.000
Combo - Limited	-0.043	0.082	-0.206	0.117	3.09	0.755
Normative - Limited	0.042	0.083	-0.122	0.207	3.00	0.750
Unlimited - Combo	0.537	0.083	0.377	0.699	0.00	0.000
Normative - Combo	0.084	0.083	-0.076	0.253	2.09	0.677
Unlimited - Normative	0.453	0.084	0.287	0.616	0.00	0.000

Malleable belief

Table SR5.4 Model output from multiple regression model for belief that empathy is malleable

condition	emmean	lower.HPD	upper.HPD
Combo	0.089	-0.035	0.205
Control	-0.016	-0.132	0.098

Limited	-0.045	-0.167	0.073
Normative	0.034	-0.088	0.159
Unlimited	-0.064	-0.186	0.055

Table SR5.5 Contrasts between model coefficients from multiple regression models for beliefs about empathy being malleable

Hypothesis	Estimate	Est.Error	CI.Lower	CI.Upper	Evid.Ratio	Post.Prob
Limited - Control	-0.028	0.087	-0.198	0.145	3.07	0.754
Unlimited - Control	-0.048	0.084	-0.213	0.120	3.05	0.753
Combo - Control	0.105	0.086	-0.064	0.275	1.44	0.589
Normative - Control	0.050	0.087	-0.120	0.222	2.82	0.738
Unlimited - Limited	-0.020	0.088	-0.194	0.151	3.06	0.754
Combo - Limited	0.132	0.088	-0.038	0.306	1.07	0.518
Normative - Limited	0.078	0.087	-0.094	0.249	2.24	0.691
Unlimited - Combo	-0.153	0.086	-0.324	0.016	0.63	0.387
Normative - Combo	-0.055	0.087	-0.231	0.120	2.76	0.734
Unlimited - Normative	-0.098	0.087	-0.268	0.074	1.74	0.635

Normative belief

Table SR5.6 Model output from multiple regression model for belief that empathy is normative

condition	emmean	lower.HPD	upper.HPD
Combo	-0.039	-0.158	0.079
Control	-0.024	-0.143	0.095
Limited	-0.035	-0.163	0.081
Normative	0.006	-0.108	0.130
Unlimited	0.095	-0.024	0.210

Table SR5.7 Contrasts between model coefficients from multiple regression models for beliefs about empathy being normative

Hypothesis	Estimate	Est.Error	CI.Lower	CI.Upper	Evid.Ratio	Post.Prob
Limited - Control	-0.010	0.087	-0.182	0.162	3.122	0.757
Unlimited - Control	0.120	0.085	-0.044	0.288	1.203	0.546
Combo - Control	-0.014	0.086	-0.183	0.153	3.230	0.764
Normative - Control	0.031	0.087	-0.143	0.201	3.157	0.759
Unlimited - Limited	0.131	0.086	-0.037	0.295	1.066	0.516
Combo - Limited	-0.004	0.086	-0.173	0.169	3.337	0.769
Normative - Limited	0.041	0.087	-0.129	0.211	2.884	0.743
Unlimited - Combo	0.135	0.083	-0.026	0.299	0.971	0.493
Normative - Combo	0.045	0.085	-0.121	0.208	2.998	0.750

Unlimited - Normative	0.090	0.086	-0.076	0.260	1.903	0.656

Empathy

Table SR5.8 Model output from multilevel regression model for single-item empathy

condition	emmean	lower.HPD	upper.HPD
Combo	-0.065	-0.201	0.062
Control	0.086	-0.044	0.220
Limited	-0.056	-0.195	0.078
Normative	0.068	-0.071	0.204
Unlimited	-0.038	-0.178	0.101

Table SR5.9 Contrasts between model coefficients from multilevel regression models for single-item empathy

Hypothesis	Estimate	Est.Error	CI.Lower	CI.Upper	Evid.Ratio	Post.Prob
Limited - Control	-0.143	0.074	-0.288	0.002	0.634	0.388
Unlimited - Control	-0.125	0.075	-0.275	0.021	1.018	0.505
Combo - Control	-0.152	0.075	-0.298	-0.007	0.533	0.348
Normative - Control	-0.019	0.077	-0.171	0.131	3.373	0.771
Unlimited - Limited	0.018	0.078	-0.134	0.172	3.417	0.774
Combo - Limited	-0.009	0.076	-0.159	0.139	3.646	0.785
Normative - Limited	0.124	0.079	-0.033	0.279	1.102	0.524
Unlimited - Combo	0.027	0.077	-0.122	0.179	3.552	0.780
Normative - Combo	0.133	0.076	-0.019	0.282	0.782	0.439

Unlimited - Normative	-0.106	0.078	-0.263	0.047	1.448	0.592

Empathic reactions

Table SR5.10 Model output from multilevel regression model for empathic reactions

condition	emmean	lower.HPD	upper.HPD
Combo	-0.061	-0.192	0.074
Control	0.124	-0.011	0.268
Limited	-0.067	-0.206	0.065
Normative	0.056	-0.081	0.192
Unlimited	-0.042	-0.180	0.092

Table SR5.11 Contrasts between model coefficients from multilevel regression models for empathic reactions

Hypothesis	Estimate	Est.Error	CI.Lower	CI.Upper	Evid.Ratio	Post.Prob
Limited - Control	-0.193	0.077	-0.338	-0.035	0.241	0.194
Unlimited - Control	-0.168	0.079	-0.324	-0.013	0.344	0.256
Combo - Control	-0.186	0.076	-0.335	-0.037	0.204	0.170
Normative - Control	-0.069	0.078	-0.222	0.084	2.524	0.716
Unlimited - Limited	0.025	0.078	-0.128	0.180	3.543	0.780
Combo - Limited	0.006	0.076	-0.139	0.152	3.619	0.783
Normative - Limited	0.123	0.077	-0.028	0.271	1.084	0.520
Unlimited - Combo	0.018	0.077	-0.131	0.173	3.633	0.784
Normative - Combo	0.117	0.077	-0.037	0.265	1.213	0.548
Unlimited - Normative	-0.098	0.079	-0.251	0.058	1.535	0.605

Empathic drivers

Table SR5.12 Model output from multiple regression model for empathic drivers

condition	emmean	lower.HPD	upper.HPD
Combo	-0.012	-0.127	0.103
Control	0.044	-0.080	0.163
Limited	-0.011	-0.127	0.111
Normative	0.054	-0.063	0.169
Unlimited	-0.074	-0.198	0.047

Table SR5.13 Contrasts between model coefficients from multiple regression models for empathic drivers

Hypothesis	Estimate	Est.Error	CI.Lower	CI.Upper	Evid.Ratio	Post.Prob
Limited - Control	-0.056	0.088	-0.232	0.112	2.67	0.727
Unlimited - Control	-0.119	0.086	-0.287	0.056	1.23	0.552
Combo - Control	-0.057	0.088	-0.230	0.110	2.67	0.727
Normative - Control	0.009	0.088	-0.164	0.178	3.34	0.770
Unlimited - Limited	-0.063	0.088	-0.234	0.110	2.38	0.704
Combo - Limited	-0.001	0.085	-0.168	0.165	3.17	0.760
Normative - Limited	0.065	0.086	-0.101	0.232	2.48	0.712
Unlimited - Combo	-0.061	0.085	-0.226	0.107	2.63	0.724
Normative - Combo	0.067	0.083	-0.093	0.230	2.63	0.725

Unlimited - Normative	-0.128	0.084	-0.296	0.040	1.02	0.505

Donation

Table SR5.14 Model output from multiple regression model for donations

condition	emmean	lower.HPD	upper.HPD
Combo	-0.046	-0.162	0.068
Control	-0.053	-0.168	0.069
Limited	0.115	-0.003	0.234
Normative	0.065	-0.053	0.182
Unlimited	-0.074	-0.194	0.041

Table SR5.15 Contrasts between model coefficients from multiple regression models for donations

Hypothesis	Estimate	Est.Error	CI.Lower	CI.Upper	Evid.Ratio	Post.Prob
Limited - Control	0.168	0.084	0.001	0.337	0.497	0.332
Unlimited - Control	-0.021	0.086	-0.190	0.146	3.144	0.759
Combo - Control	0.008	0.084	-0.154	0.170	3.386	0.772
Normative - Control	0.118	0.087	-0.050	0.290	1.404	0.584
Unlimited - Limited	-0.189	0.085	-0.358	-0.025	0.285	0.222
Combo - Limited	-0.161	0.083	-0.323	0.003	0.583	0.368
Normative - Limited	-0.050	0.084	-0.215	0.114	2.809	0.737
Unlimited - Combo	-0.029	0.084	-0.193	0.131	3.135	0.758
Normative - Combo	0.110	0.084	-0.055	0.275	1.540	0.606
Unlimited - Normative	-0.139	0.084	-0.302	0.024	0.836	0.455

covariate IAC

Table SR5.16. Summarized model output from multiple regression model with interaction between condition and IAC

	Estimate	Est.Error	l-95% CI	u-95% CI	Rhat	Bulk_ESS
Combo	-0.05	0.03	-0.10	0.01	1.00	28694
Control	-0.03	0.03	-0.09	0.02	1.00	29021
Limited	0.11	0.03	0.05	0.17	1.00	27980
Normative	0.04	0.03	-0.01	0.10	1.00	29571
Unlimited	-0.07	0.03	-0.12	-0.01	1.00	30162
IAC	0.45	0.03	0.39	0.51	1.00	11760
Control:IAC	0.02	0.04	-0.06	0.10	1.00	15074
Limited:IAC	-0.03	0.04	-0.11	0.05	1.00	15815
Normative:IAC	-0.05	0.04	-0.13	0.04	1.00	15721
Unlimited:IAC	-0.15	0.04	-0.24	-0.07	1.00	14870

Study 5

Manipulation check

Table SR6.1 Model output for beliefs about empathy being an un/limited resource

condition	emmean	lower.HPD	upper.HPD
Control	0.055	-0.060	0.161
Hasson_control	0.048	-0.063	0.162
Limited	-0.482	-0.594	-0.372
Unlimited	0.373	0.262	0.488

Table SR6.2 Hypotheses for beliefs about empathy being an un/limited resource

Hypothesis	Estimate	Est.Error	CI.Lower	CI.Upper	Evid.Ratio	Post.Prob
((conditionLimited-conditionControl)) = 0	-0.537	0.080	-0.693	-0.382	0.000	0.000
((conditionUnlimited-conditionControl)) = 0	0.319	0.082	0.160	0.481	0.002	0.002
((conditionHasson_control-conditionControl)) = 0	-0.007	0.081	-0.165	0.151	3.439	0.775
((conditionUnlimited-conditionLimited)) = 0	0.856	0.081	0.699	1.016	0.000	0.000
((conditionHasson_control-conditionLimited)) = 0	0.530	0.080	0.373	0.690	0.000	0.000
((conditionUnlimited-conditionHasson_control)) = 0	0.326	0.081	0.164	0.487	0.000	0.000

Single-item Empathy

Table SR6.3 Model output for single-item empathy

condition	emmean	lower.HPD	upper.HPD
Control	0.100	-0.059	0.259

condition	emmean	lower.HPD	upper.HPD
Hasson_control	-0.004	-0.163	0.157
Limited	-0.150	-0.316	0.006
Unlimited	0.051	-0.110	0.211

Table SR6.4 Contrasts for single-item empathy

Hypothesis	Estimate	Est.Error	CI.Lower	CI.Upper	Evid.Ratio	Post.Prob
((conditionLimited-conditionControl)) = 0	-0.250	0.072	-0.395	-0.109	0.009	0.009
((conditionUnlimited-conditionControl)) = 0	-0.049	0.072	-0.195	0.091	2.868	0.741
((conditionHasson_control-conditionControl)) = 0	-0.103	0.072	-0.244	0.040	1.481	0.597
((conditionUnlimited-conditionLimited)) = 0	0.201	0.074	0.056	0.349	0.077	0.072
((conditionHasson_control-conditionLimited)) = 0	0.147	0.074	0.001	0.293	0.519	0.342
((conditionUnlimited-conditionHasson_control)) = 0	0.054	0.073	-0.088	0.196	2.963	0.748

Table SR6.5 Model output from for single-item empathy when condition interact with group

condition	emmean	lower.HPD	upper.HPD
Control	0.094	-0.046	0.235
Hasson_control	-0.004	-0.140	0.140
Limited	-0.142	-0.288	-0.010
Unlimited	0.049	-0.089	0.188

Table SR6.6 Contrast between conditions in same context

Hypothesis	Estimate	Est.Error	CI.Lower	CI.Upper	Evid.Ratio	Post.Prob
((conditionLimited:groupIngroup-conditionControl:groupIngroup)) = 0	-0.308	0.083	-0.473	-0.147	0.004	0.004
((conditionLimited:groupIngroup-conditionHasson_control:groupIngroup)) = 0	-0.194	0.083	-0.355	-0.031	0.268	0.212
((conditionUnlimited:groupIngroup-conditionHasson_control:groupIngroup)) = 0	-0.027	0.083	-0.190	0.135	3.142	0.759
((conditionUnlimited:groupIngroup-conditionControl:groupIngroup)) = 0	-0.141	0.084	-0.304	0.022	0.878	0.468
((conditionUnlimited:groupIngroup-conditionLimited:groupIngroup)) = 0	0.167	0.083	0.004	0.330	0.506	0.336
((conditionControl:groupIngroup-conditionHasson_control:groupIngroup)) = 0	0.114	0.083	-0.051	0.274	1.444	0.591
((conditionLimited:groupOutgroup-conditionControl:groupOutgroup)) = 0	-0.162	0.083	-0.325	-0.003	0.515	0.340
((conditionLimited:groupOutgroup-conditionHasson_control:groupOutgroup)) = 0	-0.082	0.085	-0.248	0.087	2.160	0.684
((conditionUnlimited:groupOutgroup-conditionHasson_control:groupOutgroup)) = 0	0.132	0.083	-0.030	0.293	1.083	0.520
((conditionUnlimited:groupOutgroup-conditionControl:groupOutgroup)) = 0	0.052	0.083	-0.115	0.214	2.729	0.732

((conditionUnlimited:groupOutgroup-conditionLimited:groupOutgroup)) = 0	0.214	0.083	0.048	0.377	0.130	0.115
((conditionControl:groupOutgroup-conditionHasson_control:groupOutgroup)) = 0	0.080	0.084	-0.085	0.248	2.132	0.681

Table SR6.7 Contrast across conditions for same condition

Hypothesis	Estimate	Est.Error	CI.Lower	CI.Upper	Evid.Ratio	Post.Prob
((conditionLimited:groupIngroup-conditionLimited:groupOutgroup)) = 0	0.119	0.065	-0.005	0.250	0.809	0.447
((conditionUnlimited:groupIngroup-conditionUnlimited:groupOutgroup)) = 0	0.073	0.061	-0.050	0.192	2.313	0.698
((conditionControl:groupIngroup-conditionControl:groupOutgroup)) = 0	0.265	0.061	0.144	0.387	0.000	0.000
((conditionHasson_control:groupIngroup-conditionHasson_control:groupOutgroup)) = 0	0.232	0.063	0.108	0.356	0.003	0.003

Empathic Reactions

Table SR6.8 Model output for empathic reactions

condition	emmean	lower.HPD	upper.HPD
Control	0.112	-0.050	0.277
Hasson_control	0.008	-0.152	0.187
Limited	-0.126	-0.285	0.036
Unlimited	0.011	-0.152	0.177

Table SR6.9 Contrasts between interventions for empathic reactions

Hypothesis	Estimate	Est.Error	CI.Lower	CI.Upper	Evid.Ratio	Post.Prob
((conditionLimited-conditionControl)) = 0	-0.238	0.071	-0.379	-0.099	0.012	0.011
((conditionUnlimited-conditionControl)) = 0	-0.102	0.071	-0.243	0.037	1.469	0.595
((conditionHasson_control-conditionControl)) = 0	-0.104	0.074	-0.250	0.040	1.434	0.589
((conditionUnlimited-conditionLimited)) = 0	0.135	0.072	-0.005	0.278	0.663	0.399
((conditionHasson_control-conditionLimited)) = 0	0.134	0.074	-0.009	0.279	0.785	0.440
((conditionUnlimited-conditionHasson_control)) = 0	0.001	0.072	-0.140	0.142	4.074	0.803

Table SR6.10 Model output for empathic reactions when condition interacts with group

condition	emmean	lower.HPD	upper.HPD
Control	0.103	-0.034	0.247
Hasson_control	0.006	-0.134	0.146
Limited	-0.119	-0.265	0.022
Unlimited	0.007	-0.139	0.141

Table SR6.11 Contrast between conditions in same context

Hypothesis	Estimate	Est.Error	CI.Lower	CI.Upper	Evid.Ratio	Post.Prob
((conditionLimited:groupIngroup-conditionControl:groupIngroup)) = 0	-0.305	0.083	-0.468	-0.145	0.005	0.005
((conditionLimited:groupIngroup-conditionHasson_control:groupIngroup)) = 0	-0.183	0.082	-0.345	-0.025	0.291	0.225
((conditionUnlimited:groupIngroup-conditionHasson_control:groupIngroup)) = 0	-0.083	0.082	-0.242	0.079	2.101	0.678
((conditionUnlimited:groupIngroup-conditionControl:groupIngroup)) = 0	-0.205	0.082	-0.366	-0.043	0.169	0.145
((conditionUnlimited:groupIngroup-conditionLimited:groupIngroup)) = 0	0.100	0.082	-0.058	0.263	1.688	0.628
((conditionControl:groupIngroup-conditionHasson_control:groupIngroup)) = 0	0.122	0.082	-0.040	0.281	1.155	0.536
((conditionLimited:groupOutgroup-conditionControl:groupOutgroup)) = 0	-0.142	0.082	-0.302	0.020	0.731	0.422
((conditionLimited:groupOutgroup-conditionHasson_control:groupOutgroup)) = 0	-0.067	0.082	-0.229	0.091	2.468	0.712
((conditionUnlimited:groupOutgroup-conditionHasson_control:groupOutgroup)) = 0	0.086	0.081	-0.074	0.246	1.923	0.658
((conditionUnlimited:groupOutgroup-conditionControl:groupOutgroup)) = 0	0.011	0.082	-0.148	0.169	3.250	0.765
((conditionUnlimited:groupOutgroup-conditionLimited:groupOutgroup)) = 0	0.153	0.082	-0.010	0.315	0.633	0.387
((conditionControl:groupOutgroup-conditionHasson_control:groupOutgroup)) = 0	0.075	0.082	-0.090	0.237	2.274	0.695

Table SR6.12 Contrast across contexts for same condition

Hypothesis	Estimate	Est.Error	CI.Lower	CI.Upper	Evid.Ratio	Post.Prob
((conditionLimited:groupIngroup-conditionLimited:groupOutgroup)) = 0	0.133	0.063	0.013	0.255	0.393	0.282
((conditionUnlimited:groupIngroup-conditionUnlimited:groupOutgroup)) = 0	0.081	0.061	-0.039	0.201	1.874	0.652
((conditionControl:groupIngroup-conditionControl:groupOutgroup)) = 0	0.296	0.063	0.175	0.419	0.000	0.000

((conditionHasson_control:groupIngroup-conditionHasson_control:groupOutgroup)) = 0	0.249	0.062	0.130	0.372	0.000	0.000

Support Prosocial Action

Table SR6.13 Model output for support for prosocial action

condition	emmean	lower.HPD	upper.HPD
Control	0.094	-0.049	0.243
Hasson_control	0.015	-0.122	0.162
Limited	-0.067	-0.207	0.078
Unlimited	0.001	-0.138	0.147

Table SR6.14 Contrast between conditions for support for prosocial action

Hypothesis	Estimate	Est.Error	CI.Lower	CI.Upper	Evid.Ratio	Post.Prob
((conditionLimited-conditionControl)) = 0	-0.160	0.074	-0.307	-0.017	0.412	0.292
((conditionUnlimited-conditionControl)) = 0	-0.093	0.072	-0.233	0.047	1.861	0.650
((conditionHasson_control-conditionControl)) = 0	-0.076	0.073	-0.221	0.068	2.061	0.673
((conditionUnlimited-conditionLimited)) = 0	0.067	0.073	-0.074	0.211	2.677	0.728
((conditionHasson_control-conditionLimited)) = 0	0.084	0.074	-0.058	0.232	1.982	0.665
((conditionUnlimited-conditionHasson_control)) = 0	-0.017	0.074	-0.161	0.126	3.820	0.793

Table SR6.15 Model output for support of prosocial action when condition interacts with group

condition	emmean	lower.HPD	upper.HPD
Control	0.077	-0.059	0.227
Hasson_control	0.006	-0.130	0.154
Limited	-0.074	-0.215	0.070
Unlimited	-0.009	-0.146	0.139

Table SR6.16 Contrasts between conditions in same context

Hypothesis	Estimate	Est.Error	CI.Lower	CI.Upper	Evid.Ratio	Post.Prob
((conditionLimited:groupIngroup-conditionControl:groupIngroup)) = 0	-0.261	0.082	-0.422	-0.098	0.019	0.019
((conditionLimited:groupIngroup-conditionHasson_control:groupIngroup)) = 0	-0.090	0.082	-0.251	0.074	1.966	0.663
((conditionUnlimited:groupIngroup-conditionHasson_control:groupIngroup)) = 0	-0.041	0.082	-0.199	0.123	3.075	0.755
((conditionUnlimited:groupIngroup-conditionControl:groupIngroup)) = 0	-0.212	0.083	-0.378	-0.050	0.133	0.117
((conditionUnlimited:groupIngroup-conditionLimited:groupIngroup)) = 0	0.049	0.082	-0.113	0.208	2.833	0.739
((conditionControl:groupIngroup-conditionHasson_control:groupIngroup)) = 0	0.171	0.082	0.013	0.332	0.380	0.275
((conditionLimited:groupOutgroup-conditionControl:groupOutgroup)) = 0	-0.040	0.082	-0.203	0.121	3.123	0.757
((conditionLimited:groupOutgroup-conditionHasson_control:groupOutgroup)) = 0	-0.071	0.082	-0.231	0.090	2.461	0.711
((conditionUnlimited:groupOutgroup-conditionHasson_control:groupOutgroup)) = 0	0.009	0.082	-0.153	0.167	3.386	0.772
((conditionUnlimited:groupOutgroup-conditionControl:groupOutgroup)) = 0	0.040	0.083	-0.122	0.202	2.929	0.745
((conditionUnlimited:groupOutgroup-conditionLimited:groupOutgroup)) = 0	0.080	0.083	-0.080	0.244	2.034	0.670
((conditionControl:groupOutgroup-conditionHasson_control:groupOutgroup)) = 0	-0.031	0.082	-0.193	0.127	3.271	0.766

Table SR6.17 Contrasts across contexts for same condition

Hypothesis	Estimate	Est.Error	CI.Lower	CI.Upper	Evid.Ratio	Post.Prob
((conditionLimited:groupIngroup-conditionLimited:groupOutgroup)) = 0	0.079	0.061	-0.040	0.198	1.92	0.657
((conditionUnlimited:groupIngroup-conditionUnlimited:groupOutgroup)) = 0	0.049	0.059	-0.069	0.164	3.26	0.765
((conditionControl:groupIngroup-conditionControl:groupOutgroup)) = 0	0.301	0.060	0.187	0.417	0.00	0.000
((conditionHasson_control:groupIngroup-conditionHasson_control:groupOutgroup)) = 0	0.098	0.061	-0.020	0.217	1.24	0.554

IRI

Concern

Table SR6.18 Contrasts for single-item empathy when controlling for concern

Hypothesis	Estimate	Est.Error	CI.Lower	CI.Upper	Evid.Ratio	Post.Prob
(conditionLimited-conditionUnlimited) = 0	-0.148	0.067	-0.278	-0.018	0.394	0.283
(conditionLimited-conditionControl) = 0	-0.150	0.066	-0.281	-0.020	0.348	0.258
(conditionLimited-conditionHasson_control) = 0	-0.105	0.066	-0.234	0.027	1.291	0.563
(conditionUnlimited-conditionControl) = 0	-0.002	0.066	-0.128	0.128	4.588	0.821
(conditionUnlimited-conditionHasson_control) = 0	0.043	0.066	-0.085	0.173	3.464	0.776
(conditionControl-conditionHasson_control) = 0	0.045	0.066	-0.086	0.171	3.379	0.772

Table SR6.19 Contrasts for empathic reactions when controlling for concern

Hypothesis	Estimate	Est.Error	CI.Lower	CI.Upper	Evid.Ratio	Post.Prob
(conditionLimited-conditionUnlimited) = 0	-0.084	0.065	-0.213	0.042	1.963	0.662
(conditionLimited-conditionControl) = 0	-0.141	0.065	-0.269	-0.017	0.414	0.293
(conditionLimited-conditionHasson_control) = 0	-0.091	0.065	-0.220	0.036	1.725	0.633
(conditionUnlimited-conditionControl) = 0	-0.057	0.066	-0.185	0.073	3.032	0.752
(conditionUnlimited-conditionHasson_control) = 0	-0.007	0.065	-0.137	0.120	4.219	0.808
(conditionControl-conditionHasson_control) = 0	0.050	0.065	-0.075	0.180	3.399	0.773

Table SR6.20 Contrasts for support for prosocial action when controlling for concern

Hypothesis	Estimate	Est.Error	CI.Lower	CI.Upper	Evid.Ratio	Post.Prob
(conditionLimited-conditionUnlimited) = 0	-0.021	0.068	-0.154	0.113	4.01	0.800
(conditionLimited-conditionControl) = 0	-0.074	0.069	-0.207	0.060	2.36	0.702
(conditionLimited-conditionHasson_control) = 0	-0.045	0.068	-0.177	0.087	3.36	0.771

Hypothesis	Estimate	Est.Error	CI.Lower	CI.Upper	Evid.Ratio	Post.Prob
(conditionUnlimited-conditionControl) = 0	-0.052	0.066	-0.182	0.076	3.31	0.768
(conditionUnlimited-conditionHasson_control) = 0	-0.024	0.068	-0.157	0.108	3.84	0.794
(conditionControl-conditionHasson_control) = 0	0.029	0.068	-0.105	0.159	3.88	0.795

Perspective taking

Table SR6.21 Contrasts for single-item empathy when controlling for perspective taking

Hypothesis	Estimate	Est.Error	CI.Lower	CI.Upper	Evid.Ratio	Post.Prob
(conditionLimited-conditionUnlimited) = 0	-0.130	0.069	-0.263	0.005	0.749	0.428
(conditionLimited-conditionControl) = 0	-0.176	0.068	-0.312	-0.047	0.137	0.120
(conditionLimited-conditionHasson_control) = 0	-0.105	0.067	-0.238	0.030	1.266	0.559
(conditionUnlimited-conditionControl) = 0	-0.046	0.067	-0.176	0.082	3.602	0.783
(conditionUnlimited-conditionHasson_control) = 0	0.026	0.067	-0.104	0.154	3.988	0.800
(conditionControl-conditionHasson_control) = 0	0.072	0.067	-0.061	0.204	2.382	0.704

Table SR6.22 Contrasts for empathic reactions when controlling for perspective taking

Hypothesis	Estimate	Est.Error	CI.Lower	CI.Upper	Evid.Ratio	Post.Prob
(conditionLimited-conditionUnlimited) = 0	-0.061	0.068	-0.195	0.072	2.856	0.741
(conditionLimited-conditionControl) = 0	-0.163	0.067	-0.297	-0.035	0.176	0.150
(conditionLimited-conditionHasson_control) = 0	-0.088	0.067	-0.220	0.043	1.784	0.641
(conditionUnlimited-conditionControl) = 0	-0.102	0.067	-0.233	0.032	1.316	0.568
(conditionUnlimited-conditionHasson_control) = 0	-0.028	0.067	-0.161	0.105	3.855	0.794
(conditionControl-conditionHasson_control) = 0	0.075	0.067	-0.057	0.204	2.328	0.700

Table SR6.23 Contrasts for support for prosocial action when controlling for perspective taking

Hypothesis	Estimate	Est.Error	CI.Lower	CI.Upper	Evid.Ratio	Post.Prob
(conditionLimited-conditionUnlimited) = 0	-0.001	0.070	-0.138	0.133	4.15	0.806
(conditionLimited-conditionControl) = 0	-0.092	0.069	-0.229	0.044	1.65	0.623
(conditionLimited-conditionHasson_control) = 0	-0.043	0.068	-0.177	0.090	3.40	0.773
(conditionUnlimited-conditionControl) = 0	-0.091	0.069	-0.225	0.045	1.71	0.631
(conditionUnlimited-conditionHasson_control) = 0	-0.043	0.070	-0.177	0.095	3.46	0.776
(conditionControl-conditionHasson_control) = 0	0.048	0.068	-0.084	0.184	3.21	0.763

Personal distress

Table SR6.24 Contrasts for single-item empathy when controlling for personal distress

Hypothesis	Estimate	Est.Error	CI.Lower	CI.Upper	Evid.Ratio	Post.Prob
(conditionLimited-conditionUnlimited) = 0	-0.238	0.073	-0.383	-0.096	0.027	0.027
(conditionLimited-conditionControl) = 0	-0.255	0.071	-0.394	-0.117	0.007	0.007
(conditionLimited-conditionHasson_control) = 0	-0.157	0.071	-0.296	-0.017	0.351	0.260
(conditionUnlimited-conditionControl) = 0	-0.017	0.071	-0.153	0.122	3.649	0.785
(conditionUnlimited-conditionHasson_control) = 0	0.081	0.070	-0.058	0.218	2.082	0.676
(conditionControl-conditionHasson_control) = 0	0.098	0.070	-0.039	0.234	1.590	0.614

Table SR6.25 Contrasts for empathic reactions when controlling for personal distress

Hypothesis	Estimate	Est.Error	CI.Lower	CI.Upper	Evid.Ratio	Post.Prob
(conditionLimited-conditionUnlimited) = 0	-0.168	0.071	-0.305	-0.029	0.280	0.219
(conditionLimited-conditionControl) = 0	-0.244	0.071	-0.383	-0.107	0.008	0.008
(conditionLimited-conditionHasson_control) = 0	-0.144	0.071	-0.282	-0.004	0.538	0.350
(conditionUnlimited-conditionControl) = 0	-0.076	0.070	-0.210	0.061	2.301	0.697
(conditionUnlimited-conditionHasson_control) = 0	0.025	0.071	-0.116	0.165	3.727	0.788
(conditionControl-conditionHasson_control) = 0	0.101	0.070	-0.038	0.237	1.329	0.571

Table SR6.26 Contrasts for support for prosocial action when controlling for personal distress

Hypothesis	Estimate	Est.Error	CI.Lower	CI.Upper	Evid.Ratio	Post.Prob
(conditionLimited-conditionUnlimited) = 0	-0.112	0.073	-0.253	0.032	1.306	0.566
(conditionLimited-conditionControl) = 0	-0.169	0.072	-0.310	-0.029	0.253	0.202
(conditionLimited-conditionHasson_control) = 0	-0.096	0.072	-0.239	0.043	1.599	0.615
(conditionUnlimited-conditionControl) = 0	-0.057	0.072	-0.197	0.083	2.885	0.743
(conditionUnlimited-conditionHasson_control) = 0	0.016	0.072	-0.126	0.160	3.581	0.782
(conditionControl-conditionHasson_control) = 0	0.072	0.072	-0.067	0.212	2.238	0.691

Fantasy

Table SR6.27 Contrasts for single-item empathy when controlling for fantasy

Hypothesis	Estimate	Est.Error	CI.Lower	CI.Upper	Evid.Ratio	Post.Prob
(conditionLimited-conditionUnlimited) = 0	-0.202	0.070	-0.339	-0.062	0.092	0.084
(conditionLimited-conditionControl) = 0	-0.224	0.069	-0.359	-0.087	0.036	0.035
(conditionLimited-conditionHasson_control) = 0	-0.109	0.070	-0.247	0.028	1.286	0.563

Hypothesis	Estimate	Est.Error	CI.Lower	CI.Upper	Evid.Ratio	Post.Prob
(conditionUnlimited-conditionControl) = 0	-0.022	0.070	-0.160	0.113	3.913	0.796
(conditionUnlimited-conditionHasson_control) = 0	0.092	0.069	-0.042	0.228	1.658	0.624
(conditionControl-conditionHasson_control) = 0	0.115	0.070	-0.022	0.254	1.022	0.505

Table SR6.28 Contrasts for empathic reactions when controlling for fantasy

Hypothesis	Estimate	Est.Error	CI.Lower	CI.Upper	Evid.Ratio	Post.Prob
(conditionLimited-conditionUnlimited) = 0	-0.135	0.070	-0.272	0.004	0.652	0.395
(conditionLimited-conditionControl) = 0	-0.213	0.069	-0.349	-0.074	0.032	0.031
(conditionLimited-conditionHasson_control) = 0	-0.098	0.069	-0.237	0.041	1.525	0.604
(conditionUnlimited-conditionControl) = 0	-0.078	0.068	-0.211	0.056	2.217	0.689
(conditionUnlimited-conditionHasson_control) = 0	0.036	0.071	-0.104	0.172	3.542	0.780
(conditionControl-conditionHasson_control) = 0	0.114	0.070	-0.024	0.248	1.124	0.529

Table SR6.29 Contrasts for support for prosocial action when controlling for fantasy

Hypothesis	Estimate	Est.Error	CI.Lower	CI.Upper	Evid.Ratio	Post.Prob
(conditionLimited-conditionUnlimited) = 0	-0.068	0.070	-0.201	0.068	2.417	0.707
(conditionLimited-conditionControl) = 0	-0.134	0.071	-0.273	0.006	0.741	0.426
(conditionLimited-conditionHasson_control) = 0	-0.044	0.070	-0.182	0.096	3.109	0.757
(conditionUnlimited-conditionControl) = 0	-0.066	0.070	-0.201	0.071	2.544	0.718
(conditionUnlimited-conditionHasson_control) = 0	0.024	0.071	-0.114	0.165	3.996	0.800
(conditionControl-conditionHasson_control) = 0	0.090	0.072	-0.050	0.229	1.648	0.622

Full IRI-scale

Table SR6.30 Contrasts for empathy when controlling for IRI

Hypothesis	Estimate	Est.Error	CI.Lower	CI.Upper	Evid.Ratio	Post.Prob
(conditionLimited-conditionUnlimited) = 0	-0.086	0.064	-0.213	0.040	1.801	0.643
(conditionLimited-conditionControl) = 0	-0.131	0.063	-0.255	-0.009	0.529	0.346
(conditionLimited-conditionHasson_control) = 0	-0.084	0.063	-0.207	0.041	1.826	0.646
(conditionUnlimited-conditionControl) = 0	-0.045	0.063	-0.168	0.079	3.421	0.773
(conditionUnlimited-conditionHasson_control) = 0	0.002	0.064	-0.121	0.129	4.576	0.820

Hypothesis	Estimate	Est.Error	CI.Lower	CI.Upper	Evid.Ratio	Post.Prob
(conditionControl-conditionHasson_control) = 0	0.047	0.062	-0.074	0.173	3.246	0.764

Table SR6.31 Contrasts for empathic reactions when controlling for IRI

Hypothesis	Estimate	Est.Error	CI.Lower	CI.Upper	Evid.Ratio	Post.Prob
(conditionLimited-conditionUnlimited) = 0	-0.159	0.064	-0.283	-0.034	0.231	0.187
(conditionLimited-conditionControl) = 0	-0.145	0.063	-0.273	-0.021	0.323	0.244
(conditionLimited-conditionHasson_control) = 0	-0.098	0.063	-0.218	0.027	1.358	0.575
(conditionUnlimited-conditionControl) = 0	0.014	0.063	-0.110	0.141	4.454	0.816
(conditionUnlimited-conditionHasson_control) = 0	0.061	0.062	-0.060	0.186	2.784	0.735
(conditionControl-conditionHasson_control) = 0	0.046	0.063	-0.075	0.169	3.417	0.773

Table SR6.32 Contrasts for support for prosocial action when controlling for IRI

Hypothesis	Estimate	Est.Error	CI.Lower	CI.Upper	Evid.Ratio	Post.Prob
(conditionLimited-conditionUnlimited) = 0	-0.036	0.065	-0.165	0.093	3.872	0.794
(conditionLimited-conditionControl) = 0	-0.066	0.064	-0.194	0.060	2.511	0.715
(conditionLimited-conditionHasson_control) = 0	-0.035	0.065	-0.165	0.092	3.747	0.789
(conditionUnlimited-conditionControl) = 0	-0.029	0.064	-0.155	0.097	3.835	0.793
(conditionUnlimited-conditionHasson_control) = 0	0.000	0.065	-0.128	0.131	4.226	0.808
(conditionControl-conditionHasson_control) = 0	0.030	0.065	-0.098	0.158	4.124	0.804
