# Supplementary material for: Brief empathy interventions online can decrease but not increase empathic tendencies
Source: Commun Psychol. 2025 Nov 14;3:157. doi: 10.1038/s44271-025-00364-w (PMC12618501; doi:10.1038/s44271-025-00364-w)
Supplement: Supplementary file 3 — reporting summary [file 44271_2025_364_MOESM3_ESM.pdf]

nature portfolio

Corresponding author(s): Alexander Tagesson

Last updated by author(s): Oct 23, 2025

Reporting Summary

Nature Portfolio wishes to improve the reproducibility of the work that we publish. This form provides structure for consistency and transparency in reporting. For further information on Nature Portfolio policies, see our Editorial Policies and the Editorial Policy Checklist.

Statistics

For all statistical analyses, confirm that the following items are present in the figure legend, table legend, main text, or Methods section.

n/a	Confirmed	
☐	☒	The exact sample size (*n*) for each experimental group/condition, given as a discrete number and unit of measurement
☐	☒	A statement on whether measurements were taken from distinct samples or whether the same sample was measured repeatedly
☐	☒	The statistical test(s) used AND whether they are one- or two-sided<br>*Only common tests should be described solely by name; describe more complex techniques in the Methods section.*
☐	☒	A description of all covariates tested
☐	☒	A description of any assumptions or corrections, such as tests of normality and adjustment for multiple comparisons
☐	☒	A full description of the statistical parameters including central tendency (e.g. means) or other basic estimates (e.g. regression coefficient) AND variation (e.g. standard deviation) or associated estimates of uncertainty (e.g. confidence intervals)
☐	☒	For null hypothesis testing, the test statistic (e.g. *F*, *t*, *r*) with confidence intervals, effect sizes, degrees of freedom and *P* value noted<br>*Give P values as exact values whenever suitable.*
☐	☒	For Bayesian analysis, information on the choice of priors and Markov chain Monte Carlo settings
☐	☒	For hierarchical and complex designs, identification of the appropriate level for tests and full reporting of outcomes
☐	☒	Estimates of effect sizes (e.g. Cohen's *d*, Pearson's *r*), indicating how they were calculated

Our web collection on statistics for biologists contains articles on many of the points above.

Software and code

Policy information about availability of computer code

Data collection	Experiments coded with JsPsych version 7.2 and data collected using cognition.run
Data analysis	Data were analysed using R statistical language (version 4.3.1) using the brms package

For manuscripts utilizing custom algorithms or software that are central to the research but not yet described in published literature, software must be made available to editors and reviewers. We strongly encourage code deposition in a community repository (e.g. GitHub). See the Nature Portfolio guidelines for submitting code & software for further information.

Data

Policy information about availability of data

All manuscripts must include a data availability statement. This statement should provide the following information, where applicable:

- Accession codes, unique identifiers, or web links for publicly available datasets
- A description of any restrictions on data availability
- For clinical datasets or third party data, please ensure that the statement adheres to our policy

Data and accompanying scripts will be pusblished online via OSF: https://osf.io/hr6gn/

Research involving human participants, their data, or biological material

Policy information about studies with human participants or human data. See also policy information about sex, gender (identity/presentation), and sexual orientation and race, ethnicity and racism.

Reporting on sex and gender	We report on self-reported, via Prolific, males and females.<br>We did not perform any particular gender-based analysis as this was not the focus of our study. Further details found in method section in main manuscript.
Reporting on race, ethnicity, or other socially relevant groupings	Participants self-reported their ethnicity via Prolific. In study 1 and 2 we only included participants in our analyses that stated that they perceived the protagonist in the vignette as belonging to the correct outgroup (study 1) and the correct ingroup (study 2). This was done to increase the likelihood of creating the correct experimental conditions, i.e., intergroup and ingroup contexts. Further details found in method section in main manuscript.
Population characteristics	*Describe the covariate-relevant population characteristics of the human research participants (e.g. age, genotypic information, past and current diagnosis and treatment categories). If you filled out the behavioural & social sciences study design questions and have nothing to add here, write "See above."*
Recruitment	We recruited participants online via Prolific and do not envision any particular self-selection bias. All participants got monetary compensation for their work.
Ethics oversight	Swedish Ethical Review Authority

Note that full information on the approval of the study protocol must also be provided in the manuscript.

Field-specific reporting

Please select the one below that is the best fit for your research. If you are not sure, read the appropriate sections before making your selection.

☐ Life sciences ☒ Behavioural & social sciences ☐ Ecological, evolutionary & environmental sciences

For a reference copy of the document with all sections, see nature.com/documents/nr-reporting-summary-flat.pdf

Behavioural & social sciences study design

All studies must disclose on these points even when the disclosure is negative.

Study description	We tried to replicate previously succesful interventions for getting people to increase their empathy with others. All collected data are quantitative.
Research sample	All participants were recruited via Prolific using convenience samples for online participation in experiments. Both gender, age and ethnicity were provided by Prolific and self-reported on their site by participants.<br><br>Study 1 includes 745 American participants (338 females, 405 males; 370 Black, 375 White; Mage = 41, SDage = 13)<br><br>Study 2 includes 745 American participants (359 females, 384 males; 368 Black, 377 White; Mage = 39, SDage = 13)<br><br>Study 3 includes 1056 American participants (638 females, 412 males; Mage = 38, SDage = 13)<br><br>Study 4 includes 1236 American participants (621 females, 615 males; Mage = 36.9, SDage = 13)<br><br>Study 5 includes 994 American participants (510 females, 483 males; Mage = 40.1, SDage = 13),
Sampling strategy	We used convenience samples when recruiting participants. We used the statistical software R to calculate the sample sizes needed to obtain enough power. We calculated power using both the pwr-package and performed simulations to obtain different effect-sizes to deteremine adequate sample sizes.<br><br>For our studies 1 and 2 we used statisitical software R to calculate the sample size needed to obtain 95% power to find the same effect size (Cohen's d = 0.42) as reported in Hasson and colleagues' study 2.<br><br>For our studies 3 and 4 we calculated the sample size needed to obtain at least 95% power to find the same effect size as the one we found in our study 1, i.e., Cohen's d = 0.22. We oversampled in study 4 to make sure all conditions had enough participants to obtain at least power of 95%.<br><br>In study 5, we calculated the sample size needed, using both pwr-package and simulation, to obtain at least 99% power to detect the effect size found for the interaction between condition x identity in Hasson et al's study 3 (Cohen's d = 0.5).
Data collection	Participants performed experiments online and were able to use either a desktop or tablet to complete the experiment.
Timing	All data collections were completed within a couple of days from their start date.

Timing	Study 1 started July 5, 2023.<br><br>Study 2 started Oct 26 2023.<br><br>Study 3 started Oct 24 2024<br><br>Study 4 started Dec 10 2024<br><br>Study 5 started May 15 2025
Data exclusions	We excluded appoximately 15-20 participants across all five studies for failing both attention-checks, which was a pre-registered procedure at OSF.<br><br>We excluded 140 participants each from studies 1 and 2 for stating that they perceived the protagonist as belonging to another ethnic group than the one intended to create the right conditions for intergroup and ingroup contexts. This procedure was also pre-registered at OSF.
Non-participation	Across all five studies, approximately 100-130 participants revoked their consent at Prolific without stating their reason for doing so.
Randomization	Participants were randomly allocated into experimental conditions in all five studies.

Reporting for specific materials, systems and methods

We require information from authors about some types of materials, experimental systems and methods used in many studies. Here, indicate whether each material, system or method listed is relevant to your study. If you are not sure if a list item applies to your research, read the appropriate section before selecting a response.

Materials & experimental systems

n/a	Involved in the study
☒	☐ Antibodies
☒	☐ Eukaryotic cell lines
☒	☐ Palaeontology and archaeology
☒	☐ Animals and other organisms
☒	☐ Clinical data
☒	☐ Dual use research of concern
☒	☐ Plants

Methods

n/a	Involved in the study
☒	☐ ChIP-seq
☒	☐ Flow cytometry
☒	☐ MRI-based neuroimaging

Plants

Seed stocks	*Report on the source of all seed stocks or other plant material used. If applicable, state the seed stock centre and catalogue number. If plant specimens were collected from the field, describe the collection location, date and sampling procedures.*
Novel plant genotypes	*Describe the methods by which all novel plant genotypes were produced. This includes those generated by transgenic approaches, gene editing, chemical/radiation-based mutagenesis and hybridization. For transgenic lines, describe the transformation method, the number of independent lines analyzed and the generation upon which experiments were performed. For gene-edited lines, describe the editor used, the endogenous sequence targeted for editing, the targeting guide RNA sequence (if applicable) and how the editor was applied.*
Authentication	*Describe any authentication procedures for each seed stock used or novel genotype generated. Describe any experiments used to assess the effect of a mutation and, where applicable, how potential secondary effects (e.g. second site T-DNA insertions, mosiacism, off-target gene editing) were examined.*
